# Do mHealth applications improve clinical outcomes of patients with cancer? A critical appraisal of the peer-reviewed literature

**DOI:** 10.1007/s00520-019-04945-4

**Published:** 2019-07-04

**Authors:** Jemima Osborn, Anu Ajakaiye, Tim Cooksley, Christian P. Subbe

**Affiliations:** 1 Ysbyty Gwynedd, Penrhosgarnedd, Bangor, Gwynedd LL57 2PW UK; 2 The Christie NHS Foundation Trust, Wilmslow Rd, Manchester, M20 4BX UK; 3 School of Medical Sciences, Bangor University, Brigantia Building, Penrallt Road, Bangor, Gwynedd LL57 2AS UK

**Keywords:** Cancer, mHealth, Smartphone, Internet, Health-related quality of life

## Abstract

**Purpose:**

Patients undergoing systemic anti-cancer treatment experience distressing side effects, and these symptoms are often experienced outside the hospital setting. The impact of usage of cancer-related mobile health (mHealth) applications on patient-related outcomes requires investigation.

**Methods:**

A critical appraisal of the literature was performed for the following question: ‘In patients with cancer have mHealth applications been compared with usual care to examine impact on commonly used clinical outcomes’. Literature searches were undertaken with the help of a research librarian and included Medline, Cochrane Collaboration, clinical trial databases and grey searches.

**Results:**

Seventeen studies including between 12 and 2352 patients were identified and reviewed. Smartphone applications or internet portals collected data on symptoms or patient activity. Several studies showed statistically significant differences in patient-reported outcomes when symptom monitoring using mobile health application was compared to usual care. Change in mobility was the only outcome that was related directly to toxicity. Only limited data on mortality, cancer-related morbidity including complications of care, health-economic outcomes or long-term outcomes were reported.

**Conclusions:**

Studies on mHealth applications might improve aspects of symptom control in patients with cancer, but there is currently little evidence for impact on other outcomes. This requires future research in interventional studies.

## Introduction

Complications of cancer and its treatments are common [1]. Many patients will experience side effects following chemotherapy, radiotherapy or targeted therapies. These lead to morbidity and mortality as well as increased resource utilisation in the community or hospital setting. Complications of cancer and its treatments are often predictable (fever, diarrhoea, skin reactions and drug-specific effects). Education of patients might help to increase compliance with care pathways [2] especially if tailored to an individual’s needs. In the context of an increasingly digital healthcare system, it is therefore worth considering the role of mobile health applications (mHealth) for clinical care, patient education and safety of treatment.

No standardized definition of mHealth exists, but for the purpose of the Global Observatory for eHealth (GOe), mHealth or mobile health has been defined as ‘medical and public health practice supported by mobile devices, such as mobile phones, patient monitoring devices, personal digital assistants (PDAs), and other wireless devices’ [3]. There are currently 97,000 mobile health applications, and in 2017, the number of global users for these was thought to be at 3.4 billion patients [4]. The widespread use of smartphones (80% of patients [5], 95% of nurse and 99% of doctor [6]) in the UK means that mHealth applications are potentially accessible by most participants in healthcare: Healthcare professionals use smartphone applications to access risk assessment tools and scoring systems or to recap guidelines. Research on interventions based on mHealth applications suggests that they can be used to alter health related behaviours [7], such as medication adherence [8], but economic evidence for their usage is limited [9].

Patients use applications to get lifestyle advice, dietary information or practice mindfulness, yoga or other sports. Mobile health applications for patients with cancer might track deterioration [10] and support education and recovery [11–13] and have been suggested as a topic for research [14]. It is not known how mHealth applications affect patient-reported experience and patient-reported outcome measures. The latter can be generic or cancer specific. Patient-related outcomes measures are thought to be central for the understanding of effectiveness of treatments in cancer, improve patient-provider communication, patient satisfaction [15], everyday life [16] and survival [17].

In order to improve support of patients referred to the local oncology service that covers a large rural and remote area in North Wales, the authors reviewed the literature to identify mHealth application with a peer-reviewed evidence of impact on clinical outcomes that could be deployed in UK practice.

## Methods

### Study design

The review of the literature used the format of a ‘Critically appraised topic’ (CAT). CATs are standardized summaries which draw together best available evidence to answer questions based on real clinical scenarios [18]. CATs follow principles of evidence-based medicine in four steps: The authors (1) form a focused and answerable question based on a clinical encounter, (2) search for the best available evidence, (3) critically appraise the evidence for validity and clinical relevance and (4) examine the application of the results to clinical practice and future research.

### Search strategy

The search question was created in a patient–intervention–comparison–outcomes (PICO) format: ‘In patients with cancer (P) have mHealth applications (I) been compared with usual care (C) to examine impact on commonly used clinical outcomes (O)’.

Outcomes that are commonly used in cancer trials include mortality, morbidity, quality of life, usage of hospital beds, number of outpatient appointments or appointments in primary care. The context of care of patients with cancer morbidity related to treatments might be of particular interest.

A literature search was undertaken with the assistance of a research librarian. The following search string was used: (Mobile applications ‘OR’ Smartphone applications) ‘AND’ (Cancer ‘OR’ Neoplasms) followed by further searching using specific outcome measures: (‘morbidity’ OR ‘mortality’ OR ‘quality of life’ OR ‘hospital beds’ OR ‘patient safety’ OR ‘outpatient appointments’ OR ‘GP appointments’). Additionally, a search for studies using patient portals was conducted: (“Patient Portals”[Mesh]) AND (cancer or neoplasm). Identified papers were searched for further applicable references (‘snow balling’).

### Inclusion and exclusion criteria

Study criteria were agreed prior to undertaking the review: Publications up to April 2018 were included. No study pre-dating 2014 was identified. Randomized and non-randomized studies on all types of cancer including haematological malignancies were included. The review included dedicated mobile applications as well as programs that could be used on a smartphone such as web portals.

Non-patient-facing applications, research protocols, studies that did not measure clinical outcomes and studies that reported purely application feasibility were excluded.

Studies were selected by one of the investigators (JO) and confirmed by the second investigator (AA). The papers identified in the search were analysed using the following questions: Does the study address the research question, were the study methods valid in a generic oncology setting and are the results applicable to patients with cancer looked after in a clinical (vs research) setting.

Search terms were applied to Pubmed, Embase, Cochrane library and a national registry of trials (ClinicalTrials.gov).

No funding was received for the undertaking of the review.

## Results

### Identified studies

**Fig. 1:**
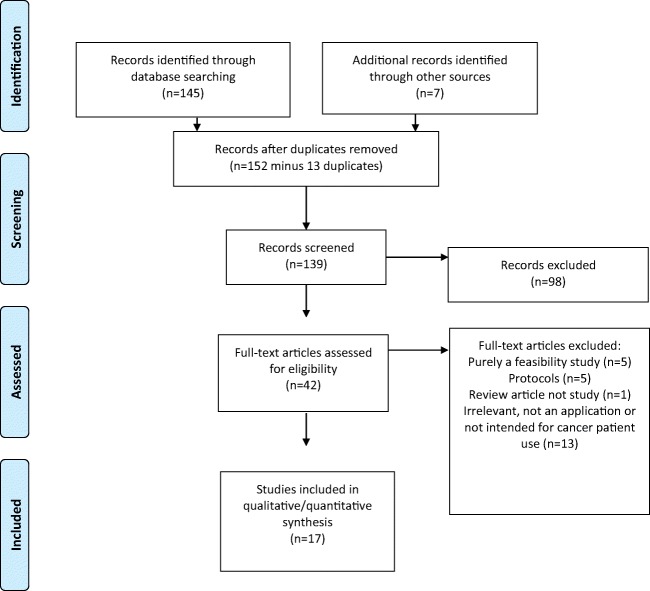
PRISMA flow diagram of literature search

The search found 139 abstracts, of which 17 fulfilled inclusion and exclusion criteria (Fig. 1). Eighty-four studies initially identified did not meet the inclusion criteria as they did not measure a patient-related outcome or were not for direct patient use.

The Cochrane Library identified a number of systematic reviews of mobile Health applications but none in the context of cancer care. The national database of clinical trials (ClinicalTrial.Gov) identified 72 trials; 20 of these were marked as ‘completed’, and two had published results in the peer-reviewed literature [19, 20].

Seventeen studies met inclusion and exclusion criteria. Sample sizes varied from 12 to 2352 patients with a median of 130 patients. Eleven of the studies had less than 100 participants. Ten of the studies were randomized controlled trials using usual care as their comparator. Patients with breast cancer were the patient group most commonly targeted (6 studies) (Tables 1, 2, and 3). Studies examined effects of custom-built smartphone applications and internet portals as well as existing messaging services [21] and patient portals [22].

**Table 1:** Studies on mHealth applications for patients with cancer

Author (year)	Country	Type of application	Number of patients	Study design	Comparator group	Patient activity	Application function
Aljabri D (2018)	USA	Internet portal	2352 patients	Retrospective cohort study	Non-adopters	Not reported	Access to clinical records
Basch E (2015)	USA	Internet portal or kiosk in hospital	766 patients; 441 intervention, 325 control	Randomized	Usual care	Symptom-checker: chemotherapy-related symptoms	Alert: e-mail to nurses for significant or worsening patient-reported symptoms
Berry D (2015)	USA	Internet portal	752 patients; 256 intervention group, 261 control group	Randomized	Usual care	Symptom-checker & quality of questionnaires	Education: information about symptoms and reporting
Denis F (2014)	France	Mobile application	42 patients	Cohort study	Clinic appointment	Symptom-checker	Alert: of oncologist
Foley N (2016)	Ireland	Mobile application	39 patients; 13 intervention group; 26 control group	Randomized	Standard leaflets	None	Education: information about breast cancer
Fortier M (2016)	USA	Mobile application	12 patients	Pilot study	n.a.	Symptom-checker: avatar interaction on reporting of pain	Education: link to information Alert
Golsteijn RHJ (2018)	Netherlands	Internet portal	478 patients; 249 intervention, 229 control	Randomized	Waiting list controlled	Life-style: exercise data	Education: exercise advice
Jibb LA (2017)	Canada	Mobile application	40 patients	Cohort study	n.a.	Symptom checker: pain measured by questionnaire	Education: link to information
Kanera I (2017)	Netherlands	Internet portal	462 patients; 231 intervention group, 231 control group	Randomized	Usual care	Life-style: physical activity & vegetable intake measured by questionnaires	Education: link to educational content
Kolb NA (2018)	USA	Automated telephone system	252 patients; 121 intervention group, 131 control group	Randomized	Usual care	Symptom-checker	Education: self-care strategies Alert: nurse practitioner review
Rosen KD (2018)	USA	Mobile application	112 patients; 57 intervention, 55 control	Randomized	Waiting list controlled	Education: 12-week mindfulness course	
Smith SK (2016)	USA	Mobile application	31 patients	Cohort study	Baseline	Symptom-checker: PTSD measured by questions	Education: link to information
Soto-Pere-De-Celis E (2018)	Mexico	Mobile application	40 patients	Cohort study	Baseline	Life-style: mobility measured by accelerometer	Alert: phone call to smartphone for review of toxicity
Sundberg K (2017)	Sweden	Mobile application	130 patients; 66 intervention group, 64 control group	Non-randomized controlled study	Historic control group	Symptom-checker: psychological distress measured by questionnaire	Alert: oncology nurse
Uhm K (2017)	Korea	Mobile application	356 patients; 179 intervention group, 177 control group	Randomized	Exercise brochure	Life-style: pedometer measuring activity	Education: goal setting for physical activity
Wheelock A (2014)	USA	Internet portal	100 patients; 59 intervention group, 41 control group	Randomized	Usual care	Symptom-checker: depression & quality of life measured by questionnaires	Alert: review by oncology nurse
Zou Q (2018)	China	Telephone chat application	426 patients; 251 intervention, 175 control	Randomized	Usual care	Symptom-checker: anxiety, pain & satisfaction measured by questionnaires	Contact with oncology team

**Table 2:** Functionality of applications, inclusion criteria, outcome measures and results of studies testing mHealth applications for patients with cancer

Author (year)	Name & function of application	Inclusion criteria	Outcome measures	Results
Aljabri D (2018)	Existing patient portal	Adult patient admitted to hospital with cancer as a primary or secondary diagnosis	Provider-reported, in-hospital adverse event; post-discharge emergency department visits and unplanned readmissions within 30 days; satisfaction by Hospital Consumer Assessment of Healthcare Providers and Systems (HCAHPS) survey.	Increased readmission rates among active adaptors of the patient portal. Self-management knowledge scores were higher among adopters vs non-adopters (univariate analyses only)
Basch E (2018)	**STAR** (Symptom Tracking and Reporting) Web-based interface for self-reporting of common symptoms associated with cancer treatment	Patients with metastatic breast, genitourinary, gynaecologic or lung cancers receiving chemotherapy stratified for experience with computers	Health-related quality of life (HRQoL measured by EQ-5D); emergency room visits, hospitalizations, survival.	Patient in the intervention group experienced less decline of HRQoL less frequent admissions to the emergency room, less hospitalization and remained on chemotherapy longer. Effects on HRQoL limited to the computer-experienced subgroup.
Berry D (2015)	Self-reported online assessment of cancer symptoms: Application facilitating patient self-monitored symptoms, education and coaching on how to report worries to clinicians.	Adult patients, any type of cancer or stage, about to start a new treatment for cancer.	Symptom distress	Fatigue, pain and physical function issues were reported significantly more often by patients in the intervention group.
Denis F (2014)	Sentinel follow-up questionnaire. Email alert sent to oncologist if patient reports red flag symptoms.	Lung cancer patients after undergone surgical excision, complete response or detectable but non-progressive lung cancer.	Compliance, easiness, anxiety and performances of web application for detecting cancer relapse	Relapse detection was on average 5 weeks earlier using sentinel follow-up. Reported better relationship with oncologist and reduced anxiety about follow-up.
Foley N (2016)	Application containing basic aetiology of breast cancer, treatment and surgical intervention information.	Female adult pre-operative patients with breast cancer	Anxiety and depression scores prior and post intervention	Higher anxiety levels in intervention groups.
Fortier M (2016)	**Pain buddy** Avatar-guided tablet application including a symptom diary, communication tool and coping strategies for symptom management. Triggers to healthcare providers for severe symptoms.	Paediatric patients aged 8–18, diagnosed with cancer, undergoing outpatient cancer treatment. One parent/guardian also invited to participate. No cognitive or developmental delay.	Feasibility, symptom frequency and compliance	Symptoms were reported and recommended coping strategies utilized. Only 4% of symptoms would have triggered an alert to healthcare professionals, most of these for pain. Good compliance and user satisfaction.
Golsteijn RHJ (2018)	**OncoActive** Computer-tailored physical activity program Providing personalized feedback with printed materials.	Patients and survivors with prostate and colorectal cancer from 17 hospitals throughout the Netherlands	Questionnaires for self-reported physical activity, fatigue, distress and quality of life. Actigraph for measurement of activity	Participants in the intervention group increased self-reported activity and improved physical functioning, fatigue and depression at 6 months.
Jibb LA (2017)	**Pain squad+** 22-item questionnaire to assess pain. Real-time reporting. Patients were contacted if they reported frequent pain and information was available from the application on how best to manage the pain.	Patients aged 12–28, undergoing cancer treatment, at least 2 months from diagnosis. Patients reported pain of 3/10 at least once in week prior to recruitment.	Primary: feasibility; secondary: effectiveness: pain intensity, pain interference, health-related quality of life, self-efficacy	Improvements in pain intensity and health-related quality of life. Satisfactory acceptability with good adherence by those who completed the study.
Kanera I (2017)	Web-based self-reporting questionnaires and modules providing education about diet, smoking cessation, physical activity, anxiety, depression and fatigue.	Adult patients who had completed primary cancer treatment at least 4 weeks prior. Patients with recurrent cancer and severe medical, psychiatric or cognitive diseases excluded.	Physical activity, vegetable consumption	Sustained increase in physical activity in the intervention group. Increased vegetable consumption in the intervention group, but results not sustained to 12 months.
Kolb NA (2018)	**SymptomCare@Home** Daily symptom monitoring by telephone. Intervention group with automated telephone delivered self-care strategies and alert of nurse practitioner for poor symptom control.	Patients beginning chemotherapy with taxane/platinum therapies as a part of a larger trial.	Severity, distress and impact on activity of neuropathic pain	Patients in the intervention group had significantly fewer days with moderate and severe symptoms, fewer days of symptom distress and a trend towards less activity interference.
Rosen KD (2018)	**Headspace** Commercially available mindfulness application	Women aged 25 or more within 5 years post breast cancer diagnosis	Functional Assessment of Cancer Therapy – Breast (FACT-B), mindfulness, and pain assessments at baseline, during 8 week intervention and at 12 weeks.	Participants in the intervention group reported higher quality of life with FACT-B and higher dispositional mindfulness.
Sundberg K (2017)	**Interaktor** Symptom questionnaire focusing on frequency and distress level, responses triggered red or yellow alerts to an oncology nurse.	Adults with localized prostate cancer, eligible for curative radiotherapy, considered physically, psychologically and cognitively fit enough to take part.	Symptoms and health-related quality of life	No difference within groups in symptoms over time but improvements between intervention and control group. In the control group after radiotherapy worse emotional functioning with more fatigue, nausea, insomnia and urinary symptoms.
Smith SK (2016)	**Cancer distress coach** PTSD symptom checker with advice on managing symptoms and information on reliable sources of support	Lymphoma, breast or prostate cancer patients, 19 years or older, active PTSD symptoms	PTSD symptoms, distress, self-efficacy, feasibility, acceptability and perceived usefulness.	The majority of patients found the application helpful. Statistically significant reduction is PCL-S score for PTSD symptoms after using the app. No change in self efficacy.
Soto-Pere-De-Celis E (2018)	**Accelerometer & application** Remote monitoring of daily steps, before and during chemotherapy, with a trigger of > 15% drop in baseline activity as an indicator of potential chemotoxicity.	Patients aged >65 years, any solid cancer, chemotherapy as first line in either metastatic or recurrent cancer.	Primary: feasibility; secondary: association of level of activity with grade of chemotherapy toxicity	High acceptability of application to patients despite limited interaction with mobile technology and low educational status. Association of low step counts with grade 3 toxicity.
Sundberg K (2017)	**Interaktor** Symptom questionnaire focusing on frequency and distress level, responses triggered red or yellow alerts to an oncology nurse.	Adults with localized prostate cancer, eligible for curative radiotherapy, considered physically, psychologically and cognitively fit enough to take part.	Symptoms and health related quality of life	No difference within groups in symptoms over time but improvements between intervention and control group. In the control group after radiotherapy worse emotional functioning with more fatigue, nausea, insomnia and urinary symptoms.
Uhm K (2017)	Pedometer and smartphone app which monitored a prescribed 12-week exercise programme. Quality of life assess at baseline and 12 weeks.	Histologically confirmed breast cancer, age 20 to 70 years, completion of primary cancer treatment including surgery, chemotherapy and/or radiotherapy.	Activity measurements, self-reported physical activity, quality of life	Physical function, physical activity, and Quality of Life scores were equally improved in both groups.
Wheelock A (2014)	**SIS-NET** Three-monthly web-based self-reported symptoms. Remote assessment by a nurse practitioner.	Patients with breast cancer after completion of acute treatment or any clinical trial adjuvant treatment (6 months post chemo, 3 months post hormonal therapy or surgery)	Time between symptom reporting and evaluation by healthcare professionals, use of healthcare resources.	Only 74% of symptoms addressed within less than 3 days. Significantly more symptoms reported by patients in the intervention group. No difference in oncology-related appointments, physician visits or medical tests.
Zou Q (2018)	Telephone and WeChat application	Symptomatic adults with uterine myoma	Hamilton Anxiety Scale before and after treatment, Visual Analogue Scale for pain during the first 24 h after treatment.	Patients in the intervention group had less preoperative and postoperative anxiety, less postoperative pain and higher treatment satisfaction.

**Table 3:** Compliance with and acceptability of mobile health application and potential sources for bias of the results

Author (year)	Compliance with usage	Acceptability of intervention	Bias
Aljabri D (2018)	Not reported	Not reported	Retrospective analysis of activity found that active inpatients were more likely to be younger, married and non-locals, had higher disease severity, and received medical treatment
Basch E (2015)	Not reported	Not reported	Computer-experienced group received weekly e-mail alerts in addition to the self-scoring of symptoms during clinic visits.
Berry D (2015)	Coaching intervention received in 374/389 patients	Not reported	Intervention group younger
Denis F (2014)	82% compliance	Patients felt ‘reassured by knowing that they were followed by their oncologist using the sentinel follow-up’.	Intervention and usual care completed in the same population.
Foley N (2016)	Not reported	Not reported	Small group with a significant proportion of participants that had low IT familiarity
Fortier M (2016)	Unable to report due to technical problems	Not reported	No formal control group
Golsteijn RHJ (2018)	Not reported	Not reported	Different outcomes in patients with different educational status and cancer type.
Jibb LA (2017)	Only reported for patients who had no technical problems. Compliance reported at 69%.	Satisfactory acceptability.	Intervention with significant delay, usage of a second dedicated phone was raised as a problem by participants
Kanera I (2017)	28% in intervention group used module on physical activity	Not reported	Higher drop-out rate at 12 months in the control group
Kolb NA (2018)	Not reported (4 patients did not use the service)	Not reported	Involvement of a dedicated nurse practitioner who followed up calls in the intervention group might be responsible for the effect.
Rosen KD (2018)	During a 12-week trial participants logged in to the application on average 18 days.	Not reported	Higher drop-out rate in the intervention group limits analysis.
Smith SK (2016)	Not reported	90% of participants endorsed at least moderate satisfaction with the Cancer Distress Coach application	No formal control group. Pilot study
Soto-Pere-De-Celis E (2018)	93% compliance	85% found device ‘easy to use’	No control group in relation to the clinical outcome and small sample size.
Sundberg K (2017)	Not reported	Not reported	Control group lower level of education
Uhm K (2017)	Self-assessed activity using a questionnaire based tool	Likert scales for overall satisfaction, information satisfaction, continuous use intention and intention to recommend with values from 3.8 to 4.3/5.	Different randomisation algorithm in different hospitals, older control group
Wheelock A (2014)	Not reported	Not reported	Combination of mHealth intervention with dedicated nurse practitioner
Zou Q (2018)	Not reported	Not reported	Randomisation procedure not reported. No data on matching of control and intervention group.

### Interventions delivered through mHealth applications

Interventions that were delivered in the studies fell into broad categories: (1) delivery of information/education in a digital format [23–25], (2) provision of lifestyle interventions such as mindfulness [19], exercise [26, 27] or consumption of vegetables [28] and (3) symptom scores ranging from pain [23] to psychological symptoms of post-traumatic stress disorder (PTSD) [24] and usually linked to a healthcare professional for escalation [29]. One study looking at detection of lung cancer relapse allowed patients to access follow-up and imaging sooner if concern was raised from reported symptoms [30].

### Reported outcomes

As per our inclusion criteria, only apps which measured a patient-related outcome were included (Table 2).

#### Patient symptoms

Outcomes were heterogeneous, largely focusing on symptoms related to cancer and reporting severity, distress or quality of life impact related to specific symptoms. Quality of life measures included disease specific [27] or generic [31] tools.

The main positive clinical outcome from usage of mHealth applications was significant improvement in pain intensity, pain interference and consequentially quality of life [23]; nausea, fatigue, urinary symptoms and emotional functioning [32]; fewer days of moderate-severe neuropathic symptoms, distress and activity interference [23]; reduction in post-traumatic stress disorder symptoms [24]; reductions in distress [33] and less severe neuropathic pain compared to usual care [34] at scheduled outpatient visits. Physical activity improved in two studies [20, 28]. As a caveat, in several studies, symptoms were more common in the intervention group [29, 33, 35].

#### Treatment toxicity

A Mexican study established a correlation between reduction in day-to-day mobility and chemotherapy toxicity in geriatric cancer patients [26]. Symptom scores could be used to optimize treatments [31].

#### Mortality

One of the studies has subsequently published long-term follow-up data from using a symptom tracking application [31] about improved mortality in a research letter [36]. The lack of detail makes evaluation of this publication challenging.

#### Health-economic outcomes

These were not explicitly evaluated, but outpatient appointments and readmissions to hospital provide some surrogate outcomes for financial impact [22, 29, 31] with one study quoting higher [22] and one lower hospitalisation rate [31].

#### Adverse effects

Adverse effects from using the applications were reported in two studies: higher readmission rates in a study of an existing provider portal [22] and increased anxiety and distress levels in an application with information about breast cancer [25].

#### Others

A single study focused on the detection of cancer relapse in lung cancer survivors [30]: the study looking at detection of lung cancer relapse using sentinel questionnaires. On average, relapses were found 5 weeks earlier than the planned follow-up visit, and there was a high sensitivity for detection in relapse, but the intervention did not identify a single relapse that was not also detected by sentinel follow-up.

### Methodological considerations

Studies had clearly documented inclusion criteria and methodology. All applications using symptom reporting used validated and peer-reviewed scales. While ten of the studies were randomized, for obvious reasons none of them were blinded. Education status and familiarity with internet/mobile technology improved outcomes [31] in one study but not in another [26].

Patients used the interventions in varying amounts, but little data were available on the ‘dosage’ of application usage. Increased usage might perceivably lead to improved outcomes. A ‘prescribed dose’ of intervention would facilitate evaluation but would be unrealistic as patients will experience symptoms in varying amounts and will therefore need their intervention in varying amounts [23]. Some measure of compliance was included in most studies whereas acceptability was only formally assessed in three studies (Table 3).

### Applicability of results to patients undergoing routine oncology care

Studies identified covered a wide range of ages and demonstrated that both young people and the older generation were comfortable using apps. Some of the used measurement tools referred to a specific malignancy, and extrapolation of results does therefore need to be with caution. Variation in sample size means that results from studies with smaller patient groups might be context sensitive and not be applicable without further testing in other clinical settings.

While self-reported outcomes may be subject to some recall bias [28], many of the applications allowed for in the moment reporting [23, 37] which is likely to have less recall bias than waiting to inform a medical practitioner in an outpatient or clinic setting.

### Safety aspects

Several of the applications described alert systems which informed a healthcare professional if further intervention was required, potentially improving patient safety and increasing communication between patient and healthcare providers. One application facilitated discussion between healthcare providers and patient by educating the patient on how best to communicate their concern prior to a clinic appointment [33]. Response to new symptoms was at times delayed: In ‘SIST-net’ 74% percent of new symptoms reported by patients were addressed by a nurse practitioner in under three working days; this was below the pre-set target of 90%, thus highlighting potential workload implications and the need to put robust failsafe mechanism in place to follow up reported symptoms [29].

## Discussion

The authors have identified a small number of mHealth applications that have been examined in clinical studies with a randomized or non-randomized control group. Studies identified were aimed at a range of different cancers and age groups. Positive impact was largely limited to improved symptom control, but several studies reported increased symptoms. Data on other outcomes including health economic measures were limited.

Our search is limited by several factors: In patients with cancer changes in clinical status, morbidity and mortality can be expected within months, but the sample size of most studies might have precluded significant numbers within the study duration. Only one of the studies examined impact on mortality [36]; however, since the longest study was only conducted for 12 months, there is at current lack of long-term data.

Friends, family, and other carers are often able to identify deviation from a patient’s normal status as a first step to facilitate calls for help. Only one study ‘pain buddy’, an avatar-based symptom dairy/pain management application, invited a family member to also engage with the application, so this is a potentially unique or underexplored feature [37].

The majority of studies identified were randomized controlled trial. Given the fast pace of innovation in digital technology, this might not be the best methodology to evaluate impact [38]. Smartphone applications are only one of the new digital ways to provide care with smart watches [39, 40] and telehealth [41, 42] offering alternatives to traditional models of care.

The reasons for the limited evidence for mHealth applications in cancer might be complex: mHealth applications are a relative new addition to the armamentarium of clinicians, but safety implications are potentially considerable. The novelty means that principles of design and implementation are not as clear as those used for pharmacological interventions. Mobile applications for medical purposes require compliance with regulations and the obligation to updating information. A review of mHealth applications for patients with cancer in Spain found that only half had been developed by healthcare organisations [48]. The potential lack of clinical input into the development might be one reason for limited clinical impact despite the considerable promise of applications to monitor toxicity [26] or even adjust chemotherapy drug dosing for safety impact [49].

The present search identified registered trials that might help for further insights into the impact of mHealth interventions in the near future: eRapid is a system for patients to ‘self-report and manage adverse events online during and after cancer treatment’. The platform has been developed with patients [43, 44]. Field testing has been completed [45], and the related randomized controlled trial is powered against symptom control but will include the number of hospital, primary care, and community contacts.

The eSMART trial will study an application for symptom management in a European multi-centre study to assist patients receiving chemotherapy for breast, colorectal, or haematological cancer [46]. PRISMS will attempt a similar intervention in an Australian trial of patients with haematological malignancies [47].

Patients with cancer are in principle willing to embrace application assisted care [50]: A survey of patients with prostate cancer found that out of 375 participants, about half were willing to use a cancer care–assisted app and 72% of these said data protection/pseudonymisation was important. A third of the participants who were not willing to use an application cited that secure data transfer and data storage were a concern.

The mHealth application opens the possibility of round-the-clock care where e-alerts generated from the app can be monitored and acted upon by a member of the cancer specialist team. In practice, out-of-hours services might not be robust enough to accommodate round-the-clock monitoring in many areas. While the ability for applications to facilitate improved communication and red flag alerting with health services, care needs to be made to ensure patients understand that the app is not a replacement for usual care but an adjunct [51].

MHealth interventions work in part through changing communication patterns between patients and their care network. Randomized controlled trials might not be the most suitable way to test complex multi-faceted interventions that are difficult to blind. Studies using patient registries might provide an alternative way to evaluate this type of intervention [52, 53].

## Conclusions

The CAT review was based on service consideration in the unit of the authors that provides care for patients in rural and remote areas in North Wales: This review found only a small number of studies measuring outcomes relevant to the PICO question despite a broad search string and multiple databases. Many of the screened studies looked exclusively at the design, feasibility and acceptance of mobile health applications, but there was a significant lack of evidence for the efficacy of utilizing patient-facing applications to improve clinically relevant outcomes. More in-depth studies are needed with larger cohorts to fully evaluate the impact of applications to improve patient outcomes.

## References

[1] Cooksley T, Rice T (2017). Emergency oncology: development, current position and future direction in the USA and UK. Support Care Cancer.

[2] Hibbard J Helen G (2014) Supporting people to manage their health : an introduction to patient activation. The King’s Fund, London

[3] World Health Organization (2011) mHealth: new horizons for health through mobile technologies: second global survey on eHealth Vol. 3, Global Observatory for eHealth series, the World Health Organisation

[4] Jahns R-G, Houck P (2013) Mobile health market - trends and figures 2013–2017. Berlin

[5] UK (2016) “Has never been more addicted to smartphones” - BBC news [internet]. [cited 2018 Jul 21]. Available from: https://www.bbc.co.uk/news/business-37468560. Accessed 21 June 2018

[6] Mobasheri MH, King D, Johnston M, Gautama S, Purkayastha S, Darzi A (2015). The ownership and clinical use of smartphones by doctors and nurses in the UK: a multicentre survey study. BMJ Innov.

[7] McKay FH, Cheng C, Wright A, Shill J, Stephens H, Uccellini M (2018). Evaluating mobile phone applications for health behaviour change: a systematic review. J Telemed Telecare.

[8] Haase J, Farris KB, Dorsch MP (2017). Mobile applications to improve medication adherence. Telemed e-Health.

[9] Iribarren SJ, Cato K, Falzon L, Stone PW (2017). What is the economic evidence for mHealth? A systematic review of economic evaluations of mHealth solutions. Mihalopoulos C, editor. PLoS One.

[10] Theile G, Klaas V, Tröster G, Guckenberger M (2017). mHealth technologies for palliative care patients at the interface of in-patient to outpatient care: protocol of feasibility study aiming to early predict deterioration of patient’s health status. JMIR Res Protoc.

[11] Nasi G, Cucciniello M, Guerrazzi C (2015). The role of mobile technologies in health care processes: the case of cancer supportive care. J Med Internet Res.

[12] Davis SW, Oakley-Girvan I (2015). mHealth education applications along the cancer continuum. J Cancer Educ.

[13] Geng Y, Myneni S (2015). Patient engagement in cancer survivorship care through mHealth: a consumer-centered review of existing mobile applications. AMIA Annu Symp Proc AMIA Symp.

[14] Nasi G, Cucciniello M, Guerrazzi C (2015). The performance of mHealth in cancer supportive care: a research agenda. J Med Internet Res.

[15] Chen J, Ou L, Hollis SJ (2013). A systematic review of the impact of routine collection of patient reported outcome measures on patients, providers and health organisations in an oncologic setting. BMC Health Serv Res.

[16] Catt S, Starkings R, Shilling V, Fallowfield L (2017). Patient-reported outcome measures of the impact of cancer on patients’ everyday lives: a systematic review. J Cancer Surviv.

[17] Gotay CC, Kawamoto CT, Bottomley A (2008). The prognostic significance of patient-reported outcomes in cancer clinical trials. Artic J Clin Oncol.

[18] Callander J, Anstey AV, Ingram JR, Limpens J, Flohr C, Spuls PI (2017). How to write a critically appraised topic: evidence to underpin routine clinical practice. Br J Dermatol.

[19] Rosen KD, Paniagua SM, Kazanis W, Jones S, Potter JS (2018). Quality of life among women diagnosed with breast cancer: a randomized waitlist controlled trial of commercially available mobile app-delivered mindfulness training. Psychooncology.

[20] Golsteijn RHJ, Bolman C, Volders E, Peels DA, de Vries H, Lechner L (2018). Short-term efficacy of a computer-tailored physical activity intervention for prostate and colorectal cancer patients and survivors: a randomized controlled trial. Int J Behav Nutr Phys Act.

[21] Zou Q, Zhang G, Liu Y (2018). Health education using telephone and WeChat in treatment of symptomatic uterine myoma with high-intensity focused ultrasound. Med Sci Monit Basic Res.

[22] Aljabri D, Dumitrascu A, Burton MC, White L, Khan M, Xirasagar S, Horner R, Naessens J (2018). Patient portal adoption and use by hospitalized cancer patients: a retrospective study of its impact on adverse events, utilization, and patient satisfaction. BMC Med Inform Decis Mak.

[23] Jibb LA, Stevens BJ, Nathan PC, Seto E, Cafazzo JA, Johnston DL (2017). Implementation and preliminary effectiveness of a real-time pain management smartphone app for adolescents with cancer: a multicenter pilot clinical study. Pediatr Blood Cancer.

[24] Smith SK, Kuhn E, O’Donnell J, Koontz BF, Nelson N, Molloy K (2018). Cancer distress coach: pilot study of a mobile app for managing posttraumatic stress. Psychooncology.

[25] Foley N, O’Connell E, Lehane E, Livingstone V, Maher B, S K (2016). PATI: patient accessed tailored information: a pilot study to evaluate the effect on preoperative breast cancer patients of information delivered via a mobile application. Breast.

[26] Soto-Perez-De-Celis E, Kim H, Rojo-Castillo MP, Sun C-L, Chavarri-Guerra Y, Navarrete-Reyes AP (2018). A pilot study of an accelerometer-equipped smartphone to monitor older adults with cancer receiving chemotherapy in Mexico. J Geriatr Oncol.

[27] Uhm KE, Yoo JS, Chung SH, Lee JD, Lee I, Kim JI (2017). Effects of exercise intervention in breast cancer patients: is mobile health (mHealth) with pedometer more effective than conventional program using brochure?. Breast Cancer Res Treat.

[28] Kanera IM, Willems RA, Bolman CAW, Mesters I, Verboon P, Lechner L (2017). Long-term effects of a web-based cancer aftercare intervention on moderate physical activity and vegetable consumption among early cancer survivors: a randomized controlled trial. Int J Behav Nutr Phys Act.

[29] Wheelock AE, Bock MA, Martin EL, Hwang J, Lou EM, Rugo HS (2015). SIS.NET: a randomized controlled trial evaluating a web-based system for symptom management after treatment of breast cancer. Cancer.

[30] Denis F, Viger L, Charron A, Voog E, Dupuis O, Pointreau Y, Letellier C (2014). Detection of lung cancer relapse using self-reported symptoms transmitted via an internet web-application: pilot study of the sentinel follow-up. Support Care Cancer.

[31] Basch E, Deal AM, Kris MG, Scher HI, Hudis CA, Sabbatini P, Rogak L, Bennett AV, Dueck AC, Atkinson TM, Chou JF, Dulko D, Sit L, Barz A, Novotny P, Fruscione M, Sloan JA, Schrag D (2016). Symptom monitoring with patient-reported outcomes during routine cancer treatment: a randomized controlled trial. J Clin Oncol.

[32] Sundberg K, Wengström Y, Blomberg K, Hälleberg-Nyman M, Frank C, Langius-Eklöf A (2017). Early detection and management of symptoms using an interactive smartphone application (Interaktor) during radiotherapy for prostate cancer. Support Care Cancer.

[33] Berry DL, Hong F, Halpenny B, Partridge A, Fox E, Fann JR, Wolpin S, Lober WB, Bush N, Parvathaneni U, Amtmann D, Ford R (2014). The electronic self report assessment and intervention for cancer: promoting patient verbal reporting of symptom and quality of life issues in a randomized controlled trial. BMC Cancer.

[34] Kolb NA, Smith AG, Singleton JR, Beck SL, Howard D, Dittus K, Karafiath S, Mooney K (2018). Chemotherapy-related neuropathic symptom management: a randomized trial of an automated symptom-monitoring system paired with nurse practitioner follow-up. Support Care Cancer.

[35] Foley NM, O’Connell EP, Lehane EA, Livingstone V, Maher B, Kaimkhani S (2016). PATI: patient accessed tailored information: a pilot study to evaluate the effect on preoperative breast cancer patients of information delivered via a mobile application. Breast.

[36] Basch E, Deal AM, Dueck AC, Scher HI, Kris MG, Hudis C, Schrag D (2017). Overall survival results of a trial assessing patient-reported outcomes for symptom monitoring during routine cancer treatment. JAMA.

[37] Fortier MA, Chung WW, Martinez A, Gago-Masague S, Sender L (2016). Pain buddy: a novel use of m-health in the management of children’s cancer pain. Comput Biol Med.

[38] Pham Q, Wiljer D, Cafazzo JA (2016). Beyond the randomized controlled trial: a review of alternatives in mHealth clinical trial methods. JMIR mHealth uHealth.

[39] Hoilett OS, Twibell AM, Srivastava R, Linnes JC (2018) Kick LL: A smartwatch for monitoring respiration and heart rate using photoplethysmography. Conf Proc IEEE Eng Med Biol Soc 2018:3821–3824 10.1109/EMBC.2018.8513356 PMC6448578 30441198

[40] Pope Z, Zeng N, Zhang R, Lee H, Gao Z (2018). Effectiveness of combined smartwatch and social media intervention on breast cancer survivor health outcomes: a 10-week pilot randomized trial. J Clin Med.

[41] Cox A, Lucas G, Marcu A, Piano M, Grosvenor W, Mold F, Maguire R, Ream E (2017). Cancer survivors’ experience with telehealth: a systematic review and thematic synthesis. J Med Internet Res.

[42] Williams OE, Elghenzai S, Subbe C, Wyatt JC, Williams J (2017). The use of telemedicine to enhance secondary care: some lessons from the front line. Futur Hosp J.

[43] Avery KNL, Richards HS, Portal A, Reed T, Harding R, Carter R, Bamforth L, Absolom K, O’Connell Francischetto E, Velikova G, Blazeby JM (2019). Developing a real-time electronic symptom monitoring system for patients after discharge following cancer-related surgery. BMC Cancer.

[44] Holch P, Warrington L, Bamforth LCA, Keding A, Ziegler LE, Absolom K, Hector C, Harley C, Johnson O, Hall G, Morris C, Velikova G (2017). Development of an integrated electronic platform for patient self-report and management of adverse events during cancer treatment. Ann Oncol.

[45] Warrington L, Absolom K, Holch P, Gibson A, Clayton B, Velikova G (2019). Online tool for monitoring adverse events in patients with cancer during treatment (eRAPID): field testing in a clinical setting. BMJ Open.

[46] Maguire R, Fox PA, McCann L, Miaskowski C, Kotronoulas G, Miller M, Furlong E, Ream E, Armes J, Patiraki E, Gaiger A, Berg GV, Flowerday A, Donnan P, McCrone P, Apostolidis K, Harris J, Katsaragakis S, Buick AR, Kearney N (2017). The eSMART study protocol: a randomised controlled trial to evaluate electronic symptom management using the advanced symptom management system (ASyMS) remote technology for patients with cancer. BMJ Open.

[47] Breen S, Ritchie D, Schofield P, Hsueh Y, Gough K, Santamaria N (2015). The Patient Remote Intervention and Symptom Management System (PRISMS) – a telehealth- mediated intervention enabling real-time monitoring of chemotherapy side-effects in patients with haematological malignancies: study protocol for a randomised controlled trial. Trials.

[48] Collado-Borrell R, Escudero-Vilaplana V, Ribed-Sánchez A, Ibáñez-García S, Herranz-Alonso A, Sanjurjo-Sáez M (2016). Smartphone applications for cancer patients; what we know about them?. Farm Hosp.

[49] Weaver A, Love SB, Larsen M, Shanyinde M, Waters R, Grainger L, Shearwood V, Brooks C, Gibson O, Young AM, Tarassenko L (2014). A pilot study: dose adaptation of capecitabine using mobile phone toxicity monitoring — supporting patients in their homes. Support Care Cancer.

[50] Kessel KA, Vogel MM, Kessel C, Bier H, Biedermann T, Friess H (2017). Mobile health in oncology: a patient survey about app-assisted cancer care. JMIR mHealth uHealth.

[51] Subbe CP, Øvretveit J, Quinn N, Wyatt JC (2019). DIGITAL TECHNOLOGY: opportunities and barriers for usage of personal health records in hospital – report from a ­workshop of the Health Informatics Unit at the Royal ­College of Physicians. Futur Hosp J.

[52] Buccheri S, Sarno G, Fröbert O, Gudnason T, Lagerqvist B, Lindholm D, Maeng M, Olivecrona G, James S (2019). Assessing the nationwide impact of a registry-based randomized clinical trial on cardiovascular practice. Circ Cardiovasc Interv.

[53] Sundh J, Bornefalk-Hermansson A, Ahmadi Z, Blomberg A, Janson C, Currow DC, McDonald CF, McCaffrey N, Ekström M (2019) REgistry-based randomized controlled trial of treatment and Duration and mortality in long-term OXygen therapy (REDOX) study protocol. BMC Pulm Med 19(1):50 10.1186/s12890-019-0809-7 PMC6390558 30808321

